# Serum inflammatory markers nt-probnp, hs-crp and il-6 predict disease severity and mortality in severe community-acquired pneumonia: A propensity score matching study

**DOI:** 10.5937/jomb0-58952

**Published:** 2026-01-06

**Authors:** Tong Liu, Wei Xi, Bayaer Wulijie, Lingyun Qiu, Jianjun Shuai, Fan Yang, Xingang Wang, Junwei Zhang

**Affiliations:** 1 Xinjiang Uygur Autonomous Region Hospital of Traditional Chinese Medicine, Department of Medical Imaging, Urumqi, Xinjiang, 830000, China

**Keywords:** community-acquired pneumonia, severe pneumonia, NT-proBNP interleukin, CT, vanbolnički stečena pneumonija, teška pneumonija, NT-proBNP interleukin, CT

## Abstract

**Background:**

This study aimed to evaluate the involvementof serum inflammatory markers— N-terminal pro-brainnatriuretic peptide (NT-proBNP), hypersensitive-C reactiveprotein (hs-CRP), and interleukin-6 (IL-6) - in the pathological progression of severe community-acquired pneumonia (SCAP), examine their association with computedtomography (CT) scores, and assess their combined utilityfor diagnosis and outcome prediction.

**Methods:**

We performed a propensity score-matched retrospective cohort study involving 164 SCAP patients(research group) and 164 age- and sex-matched healthycontrols (control group) enrolled between March 2024 andJanuary 2025. Serum NT-proBNP hs-CRP and IL-6 concentrations were quantified by enzyme-linked immunosorbent assay (ELISA), while chest computed tomography(CT) manifestations were evaluated using the AcuteExacerbation of Idiopathic Pulmonary Fibrosis (AE-IPF)scoring system. Comparative analyses of inflammatorymarkers and CT imaging findings were conducted, withsubsequent correlation studies, receiver operating characteristic (ROC) curve analysis, and multivariate regressionmodeling to determine their relationship with in-hospitalmortality.

**Results:**

Following propensity score matching, demographic characteristics were well-balanced between groups (standardized mean differences &lt;0.1). SCAP patientsdemonstrated significantly elevated serum levels of NTproBNP hs-CRP and IL-6 (P&lt; 0.01), along with higher CTscores than controls. Strong positive correlations wereobserved between inflammatory marker concentrationsand CT scores (P&lt; 0.01). The combined model outperformed individual biomarkers or CT alone in diagnosingSCAP (AUC 0.934, 95%CI 0.910 -0.959; P&lt; 0.001) andpredicting mortality (AUC 0.839, 95%CI 0.759-0.919;P&lt; 0.001). Multivariate analysis identified the elevation ofthese biomarkers as independent predictors of mortality inSCAP patients (P&lt; 0.01).

**Conclusions:**

NT-proBNP hs-CRP and IL-6 play pivotal rolesin promoting SCAP progression by driving inflammatorycascades and pulmonary tissue injury. The integratedassessment of these biomarkers with CT scoring significantly improves disease monitoring and prognostic assessment accuracy, potentially guiding individualized antiinflammatory interventions in SCAP management.

## Abstract (sr)

**Uvod:**

Cilj ove studije bio je da se proceni učešće serumskihinflamatornih markera - N-terminal pro-brain natriureticpeptide (NT-proBNP), hypersensitive-C reactive protein(hs-CRP) i interleukina-6 (IL-6) - u patološkoj progresijiteške vanbolničke pneumonije (SCAP), ispita njihova povezanost sa rezultatima kompjuterizovane tomografije (CT) iproceni njihova kombinovana korisnost za dijagnozu i predviđanje ishoda.

**Metode:**

Sproveli smo retrospektivnu kohortnu studiju uparenu sa propenzionističkim skorom, u kojoj je učestvovalo164 pacijenta sa skronealnom AP (istraživačka grupa) i 164zdrave kontrolne osobe uparene po starosti i polu (kontrolnagrupa), uključene između marta 2024. i januara 2025.godine. Koncentracije serumskih NT-proBNP hs-CRP i IL-6kvantifikovane su enzimski povezanim imunosorbentnimtestom (ELISA), dok su manifestacije kompjuterizovanetomografije (CT) grudnog koša procenjene korišćenjem sistema bodovanja za akutnu egzacerbaciju idiopatske plućnefibroze (AE-IPF). Sprovedene su uporedne analize inflamatornih markera i nalaza CT snimanja, nakon čega su usledilestudije korelacije, analiza ROC krive (recover operatingcharacteristic) i multivarijantno regresiono modeliranje kakobi se utvrdila njihova veza sa smrtnošću u bolnici.

**Rezultati:**

Nakon uparivanja propenzionističkih rezultata,demografske karakteristike su bile dobro izbalansirane između grupa (standardizovane srednje razlike &lt; 0,1). Pacijenti sa SCAP-om pokazali su značajno povišene serumskenivoe NT-proBNP hs-CRP i IL-6 (P&lt; 0,01), zajedno sa višimKT rezultatima u odnosu na kontrolnu grupu. Primećene sujake pozitivne korelacije između koncentracija inflamatornih markera i KT rezultata (P&lt; 0,01). Kombinovani modelje nadmašio pojedinačne biomarkere ili samo KT u dijagnostikovanju SCAP-a (AUC 0,934, 95%CI 0,910-0,959;P&lt; 0,001) i predviđanju mortaliteta (AUC 0,839, 95 %CI0,759 -0,919; P&lt; 0,001). Multivarijantna analiza je identifikovala povišenje ovih biomarkera kao nezavisne prediktore mortaliteta kod pacijenata sa SCAP-om (P&lt; 0,01).

**Zaključak:**

NT-proBNP hs-CRP i IL-6 igraju ključnu ulogu upodsticanju progresije dijabetesa iz jajnika i papileme (SCAP)pokretanjem inflamatornih kaskada i oštećenja plucnogtkiva. Integrisana procena ovih biomarkera pomoću CT skoraznačajno poboljšava praćenje bolesti i tačnost prognostičkeprocene, potencijalno usmeravajući individualizovane antiinflamatorne intervencije u lečenju dijabetesa iz jajnika ipapileme.

## Introduction

Severe community-acquired pneumonia (SCAP) represents a critical clinical entity characterized by acute lower respiratory tract infection, exhibiting persistently elevated global morbidity and mortality rates [1]. Current epidemiological evidence demonstrates that SCAP constitutes 10%-20% of hospitalized community-acquired pneumonia (CAP) cases [2], with severe cases demonstrating alarmingly high 30-day mortality rates ranging from 30% to 50%, particularly affecting vulnerable populations including the elderly, immunocompromised individuals, and patients with chronic comorbidities such as chronic obstructive pulmonary disease and diabetes mellitus [3]. Despite these concerning statistics, the molecular mechanisms driving SCAP pathogenesis remain incompletely understood, underscoring the pressing need to identify reliable biomarkers to enhance early risk prediction, facilitate dynamic disease monitoring, and guide personalized therapeutic interventions.

Emerging research has increasingly highlighted the pivotal involvement of inflammatory markers in SCAP pathophysiology [4]. N-terminal pro-brain natriuretic peptide (NT-proBNP) is an important myocardial inflammatory factor. Previous studies mainly focused on the role of NT-proBNP in cardiovascular diseases, but recent studies have found that NT-proBNP can stimulate endothelial cells to express adhesion molecules, promote leukocyte adhesion and infiltration, and accelerate the progress of pulmonary inflammation [5]. Hypersensitive-C reactive protein (hs-CRP) is one of the most sensitive systemic inflammatory markers in the body, including pyrexia induction, neutrophil mobilization, and acute-phase response, yet its dysregulated expression may precipitate deleterious cytokine storm syndromes [6]. The pleiotropic cytokine IL-6, operating downstream of hs-CRF, not only mediates acute inflammatory processes but also contributes to disease complications such as pulmonary fibrosis and coagulopathy [7]. While existing literature has examined individual inflammatory markers in isolation [8] [9], the synergistic or antagonistic effects of NT-proBNP hs-CRP and IL-6 across different SCAP stages, as well as their dynamic relationship with organ dysfunction, remain poorly understood. Additionally, the potential utility of these mediators as objective biomarkers for radiologic severity assessment—potentially overcoming inherent limitations of conventional computed tomography (CT) scoring systems including interobserver variability and radiation concerns—warrants further investigation.

To bridge these knowledge gaps, our study employs an innovative approach combining comprehensive serum inflammatory profiling with advanced quantitative radiographic evaluation, utilizing propensity score matching (PSM) methodology. This integrated strategy will enable precise characterization of the temporal expression dynamics of NT-proBNP hs- CRP and IL-6 throughout SCAP progression, while establishing novel correlations with CT-based severity indices. The findings may yield transformative insights for developing early warning tools, refining disease surveillance paradigms, and guiding precision antiinflammatory therapies in SCAP management. Furthermore, this work may pioneer the clinical implementation of combined molecular-imaging biomarker panels, representing a significant advancement with great scientific value and significant social implications. To our knowledge, this is the first PSM study integrating AE-IPF scoring with NT-proBNP/hs-CRP/IL-6 profiling to predict SCAP outcomes.

## Materials and methods

### Study design

We designed a PSM study. Based on sample size calculations performed using G*Power software, we enrolled 164 patients with SCAP admitted to our institution between March 2024 and January 2025 as the research group. Sample size was calculated in G*Power 3.1 based on: α=0.05, power=90%, effect size=0.3 (Cohen's f), yielding 154 patients per group; 10% attrition led to 164 per group. A PSM [PSM was performed using a 1:1 nearest-neighbor algorithm with a caliper of 0.1, matching on age, sex, body mass index (BMI), smoking status, alcohol consumption, and comorbidities (diabetes, hypertension, coronary heart disease, hyperlipidemia)] was performed to select 164 healthy individuals undergoing physical examinations during the same period as the control group. Inclusion Criteria: (1) Age 60 years; (2) Meeting the established diagnostic criteria for SCAP [10] with community-onset of symptoms; (3) Complete medical records available; (4) Completion of non-contrast chest CT imaging after admission. Exclusion Criteria: (1) Coexisting pulmonary pathologies including tuberculosis or silicosis; (2) Comorbid malignancies; (3) Significant hepatic/renal dysfunction or other severe systemic diseases; (4) Documented cognitive impairment or psychiatric disorders.

### Ethical statement

This study was approved by the Ethics Committee of our hospital. As this was a retrospective study using existing medical records (No. 2024022l), the hospital waived the requirement for written informed consent. All patient data were anonymized to ensure full protection of privacy.

### Laboratory tests

Fasting venous blood samples (3-5 mL) were collected from patients in the early morning. Serum was isolated by centrifugation at 3000 r/min for 15 minutes, and hs-CRP and IL-6 levels were quantified using enzyme-linked immunosorbent assay (ELISA) kits (Wuhan Betterarray Biotechnology Co., Ltd.) following the manufacturer's protocols. ELISA kits had intra-assay CV<8% and inter-assay CV<12% (manufacturer's data). Sensitivity: hs-CRP (2.1 pg/mL), IL-1β (1.0 pg/mL), IL-6 (0.5 pg/mL). Quality control samples were included in each assay batch. Serum NT-proBNP was detected by chemiluminescence immunoassay analyzer (Roche cobas e 602).

### CT scoring

Completion of non-contrast chest CT within 24 hours of admission. Two independent radiologists evaluated patients' CT scans and assigned scores based on the Acute Exacerbation of Idiopathic Pulmonary Fibrosis (AE-IPF) criteria. Lesions were scored as: 0 = absent, 1=<25% lobe involvement, 2=25-50%, 3 = 50-75%, 4 = >75%. Total score = sum of scores for all six lobes (max 24) [11]. Interrater agreement was excellent (intraclass correlation coefficient [ICC] = 0.93 for total scores; Cohen's = 0.88 for lesion classification). All ICC values >0.8 met reliability thresholds.

### Outcome measures

Serum NT-proBNP/hs-CRP/IL-6 levels, along with CT scores, were compared between the research and control groups. In-hospital mortality was recorded, and the association between biomarker levels, CT scores, and mortality was analyzed.

### Statistical analysis

Data were analyzed using SPSS 25.0 (IBM, USA). Categorical variables are presented as n (%) and compared using the chi-square test. Continuous variables were tested for normality with the Shapiro-Wilk test. Normally distributed data are expressed as mean ± standard deviation and analyzed using independent t-tests, while non-normally distributed data are reported as median (interquartile range) and compared via the Mann-Whitney U test. Bonferroni correction was applied for multiple comparisons involving inflammatory markers. Pearson's correlation was used for correlation analysis, and diagnostic performance was assessed using receiver operating characteristic (ROC) curves. A P-value<0.05 was considered statistically significant.

## Results

### Baseline characteristics after PSM

Following PSM, baseline characteristics including age, gender, smoking status, and alcohol consumption showed no statistically significant differences between the research and control groups (*P*>0.05). The standardized mean differences (SMD) for all variables were below 0.1, indicating excellent balance between the two cohorts (Table 1).

**Table 1:** Baseline data table after PSM. Note: Variables matched in PSM: age, sex, BMI, smoking, alcohol, diabetes, hypertension, coronary heart disease, hyperlipidemia.

	Control group	Research group	SMD	t or χ^2^	P
Age	67.53±8.05	68.27±7.97	0.084	0.834	0.405
Male	100 (60.98)	94 (57.32)	0.056	0.454	0.500
BMI (kg/m^2^)	22.46±2.32	22.34±2.70	0.059	0.437	0.663
Comorbid diabetes			0.055	0.311	0.577
yes	34 (20.73)	30 (18.29)			
no	130 (79.27)	134 (81.71)			
Comorbid hypertension			0.013	0.016	0.900
yes	42 (25.61)	43 (26.22)			
no	122 (74.39)	121 (73.78)			
Comorbid coronary heart disease			0.025	0.023	0.879
yes	26 (15.85)	25 (15.24)			
no	138 (84.15)	139 (84.76)			
Comorbid hyperlipidemia			0.035	0.125	0.724
yes	19 (11.59)	17 (10.37)			
no	145 (88.41)	147 (89.63)			
Length of stay in ICU (d)	16.80±6.91	17.10±7.03	0.083	0.380	0.704
Mechanical ventilation time (h)	258.04±88.96	255.55±43.23	0.039	0.322	0.748
Heart rate (beats/min)	91.87±10.09	92.94±10.40	0.084	0.943	0.346

### Comparison of inflammatory markers and CT scores

**Figure 1:**
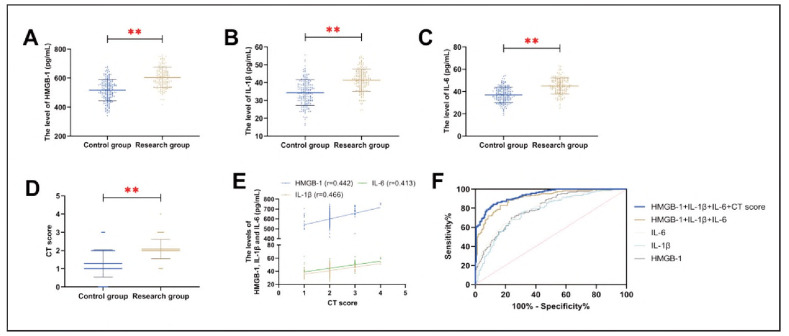
Expression differences of NT-proBNP hs-CRP and IL-6 in SCAP A: comparison of NT-proBNP B: comparison of hs-CRP C: comparison of IL-6, D: comparison of CT scores between the two groups. E: Correlation of NT-proBNP hs-CRP IL-6 and CT score in the research group. D: ROC curve of NT-proBNP hs-CRP IL-6, and CT score for the diagnosis of SCAP occurrence. **P < 0.01.

The research group demonstrated significantly elevated serum levels of NT-proBNP hs-CRP and IL-6 compared to controls (*P*<0.01), suggesting their potential role in SCAP progression. Consistent with these findings, CT severity scores were also significantly higher in the research group (*P*<0.01). Rearson correlation analysis identified significant positive correlations among NT-proBNR hs-CRR IL-6 levels, and CT scores in the research group (*P*<0.01). ROC curve analysis demonstrated that the combined detection of NT-proBNR hs-CRR and IL-6 [Log (R) = - 20.212 + 0.015xNT-proBNR + 1.315xhs-CRR +0.163xIL-6] yielded a diagnostic sensitivity of 88.41% and specificity of 78.05% for SCAP (*P*<0.001, AUC = 0.9055). When combined with CT score [Log (R) = 22.446 + 0.017xNT-proBNR + 1.032 xhs-CRR+0.151 x IL-6 + 1.852 xCT score], the diagnostic sensitivity and specificity increased to 83.54% and 88.41%, respectively (*P*<0.01, AUC = 0.9343) (Figure 1 and Table 2).

**Table 2:** Diagnostic efficacy of NT-proBNR hs-CRR IL-6, and CT score for SCAP.

	AUC	95%CI	* P *	Cut-off	Sensitivity (%)	Specificity (%)
NT-proBNR	0.7989	0.7521 to 0.8457	<0.001	>566.70	70.73	75.00
hs-CRR	0.7713	0.7206 to 0.8220	<0.001	>3.76	75.61	69.51
IL-6	0.7917	0.7435 to 0.8399	<0.001	>40.46	76.22	68.29
NT-proBNR+hs-CRR+IL-6	0.9053	0.8737 to 0.9369	<0.001	>0.4332	88.41	78.05
NT-proBNR+hs-CRR+IL-6+ CT score	0.9343	0.9098 to 0.9589	<0.001	>0.5891	83.54	88.41

### Association of T-proBNP, hs-CRP, IL-6, and CT scores with in-hospital mortality in SCAP patients

**Figure 2:**
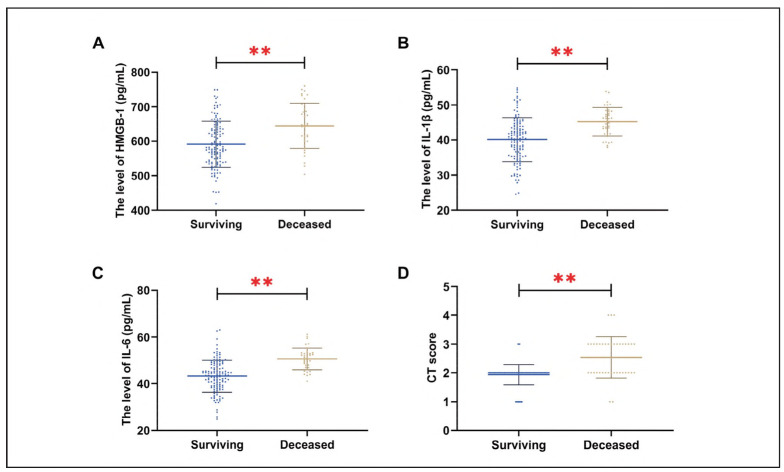
Comparison of NT-proBNR hs-CRR IL-6, and CT scores between patients who died and those who survived. A: comparison of NT-proBNR B: comparison of hs-CRR C: comparison of IL-6, D: comparison of CT scores between the two groups. **R < 0.01.

During the treatment period, 39 patients (23.78%) succumbed to the disease. Deceased patients exhibited significantly elevated levels of NT-proBNR hs-CRR and IL-6, along with higher CT scores, compared to survivors (*P*<0.01) (Figure 2).

### Impact of NT-proBNP, hs-CRP, IL-6, and CT scores on in-hospital mortality in SCAP patients

A multivariate logistic regression analysis was conducted, with patient survival status as the dependent variable (0 = survived, 1 = deceased) and NT-proBNR hs-CRR IL-6, and CT score as independent variables. The analysis revealed that elevated NT-proBNR hs-CRR and IL-6 levels, as well as higher CT scores, were independent risk factors for in-hospital mortality in SCAR patients (R<0.01) (Table 3).

**Table 3:** Effect of NT-proBNR hs-CRR IL-6, and CT on in-hospital mortality in SCAP.

	B	S.E.	Wals	Sig	OR	95%CI
NT-proBNR	0.034	0.004	11.148	<0.001	1.004	1.001-1.012
hs-CRR	0.900	0.470	3.970	0.042	2.459	1.164-6.172
IL-6	0.142	0.042	11.745	<0.001	1.153	1.063-1.251
CT score	1.238	0.581	4.545	0.033	3.448	1.105-10.757
Constant	-16.827	3.343	25.342	<0.001		

### Predictive performance of NT-proBNP, hs-CRP, IL-6, and CT score for in-hospital mortality

**Figure 3:**
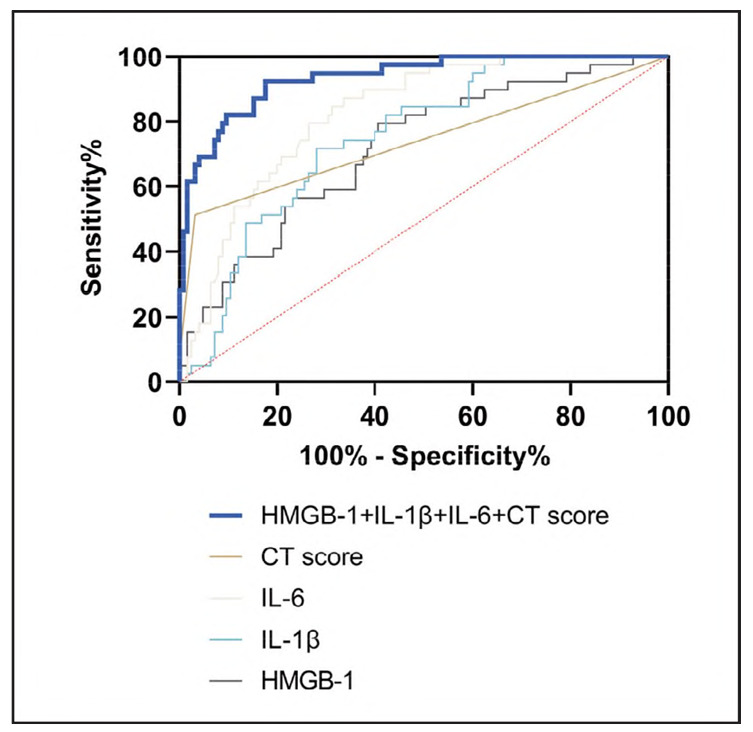
ROC curves for NT-proBNR hs-CRR IL-6, and CT Score for the diagnosis of in-hospital mortality in SCAP.

To evaluate the predictive value of these biomarkers for in-hospital mortality, ROC curve analysis was conducted using deceased patients as positive cases and survivors as negative controls. The results demonstrated that NT-proBNR hs-CRR IL-6, and CT scores individually exhibited AUC values of 0.7173, 0.7514, 0.8252, and 0.7402, respectively, in predicting in-hospital mortality among SCAP patients. Notably, the combination of all four indicators [Log (P) = -16.827 + 0.034xNT-proBNP + 0.900xhs-CRP+0.142xIL-6 + 1.238xCT score] significantly improved predictive performance, achieving an AUC of 0.9348, with a sensitivity of 71.79%, a specificity of 85.60%. The logistic regression model showed good fit (Hosmer-Lemeshow test: χ^2^ = 7.642, *P*=0.469) (Figure 3 and Table 4).

**Table 4:** Diagnostic efficacy of NT-proBNR hs-CRR IL-6, and CT Score for in-hospital mortality in SCAP.

	AUC	95%CI	* P *	Cut-off	Sensitivity (%)	Specificity (%)
NT-proBNR	0.7173	0.6274 to 0.8073	<0.001	>590.00	79.49	59.20
hs-CRR	0.7512	0.6724 to 0.8299	<0.001	>4.32	71.79	72.00
IL-6	0.8252	0.7595 to 0.8910	<0.001	>45.25	87.18	66.40
CT score	0.7402	0.6357 to 0.8447	<0.001	>2.50	51.28	96.80
NT-proBNR+hs-CRR+IL-6 +CT score	0.8392	0.7590 to 0.9194	<0.001	>0.2485	71.79	85.60

## Discussion

The pathological progression of SCAP is characterized by a complex interplay of inflammatory cascades, with NT-proBNP/hs-CRP/IL-6 emerging as pivotal pro-inflammatory markers that may play a crucial role in disease pathogenesis. Our findings demonstrate significantly elevated serum concentrations of NT-proBNP hs-CRP and IL-6 in SCAP patients, showing positive correlations with radiographic severity as assessed by CT scoring. Notably, these inflammatory markers, both individually and in combination with CT scores, serve as independent predictors of in-hospital mortality. These observations not only underscore the central involvement of inflammatory cytokines in SCAP pathophysiology but also provide clinicians with a potential multidimensional tool for assessing disease severity.

NT-proBNP functioning as a late-phase inflammatory mediator, appears to potentiate pulmonary damage in SCAP through two mechanisms. First, through its interaction with TLR4 and the receptor for advanced glycation end products (RAGE), NT-proBNP perpetuates macrophage and neutrophil activation, triggering the excessive release of reactive oxygen species (ROS) and proteolytic enzymes that ultimately lead to alveolar epithelial cell apoptosis and interstitial edema [12]. Second, it increases endothelial cell permeability, exacerbating endothelial cell permeability and subsequent inflammatory cell infiltration [13]. The significant positive correlation observed between NT-proBNP levels and CT scores (r=0.48, *P*<0.01) in our study supports its role in amplifying parenchymal injury, consistent with previous reports. For instance, Ziaie N et al. [14] showed that NT-proBNP was significantly correlated with chest CT hyperemia imaging features in COVID-19 patients.

Hs-CRP as a core regulator of innate immunity, orchestrates inflammatory responses by activating the NF- B signaling pathway, thereby stimulating the production of downstream effectors including IL-6 and tumor necrosis factor-α (TNF-α), creating a self-perpetuating inflammatory cascade [15]. In this study, the robust correlation between hs-CRP and IL-6 levels underscores their synergistic contribution to SCAP pathogenesis. Beyond its pro-inflammatory effects, IL-6 promotes a pro-thrombotic state via STAT3-mediated upregulation of fibrinogen synthesis and platelet activation, potentially predisposing to microvascular thrombosis and multiple organ dysfunction [16]. Furthermore, elevated IL-6 cells may disrupt immune homeostasis by impairing regulatory T cell (Treg) function [17]. These mechanisms collectively account for the observed association between elevated hs-CRP/IL-6 levels and increased in-hospital mortality in our patient population. This finding is in agreement with the experimental results of Sladojević M et al., [18] who demonstrated that hs-CRP and IL-6 are the main observed indicators of disease progression in patients with multiple sclerosis.

On the other hand, the observed correlations among NT-proBNP hs-CRP IL-6, and CT scores highlight the potential for integrating molecular techniques with imaging diagnostics in SCAP. CT scoring, as a quantitative measure of pulmonary parenchymal injury, demonstrates a significant positive association with these inflammatory markers, reinforcing the mechanistic connection between systemic inflammation and structural lung damage. Two key pathophysiological pathways may explain this relationship: (1) inflammatory cytokine-mediated disruption of the alveolar-capillary barrier, resulting in exudative changes visible as ground-glass opacities or consolidations on CT imaging [19], and (2) neutrophil extracellular trap (NET) formation and protease release triggering pulmonary tissue necrosis, manifesting radiologically as cavitary lesions or fibrosis [20]. Our integrative approach combining inflammatory markers with CT assessment achieved superior diagnostic accuracy for SCAP (AUC = 0.9343) and mortality prediction (AUC = 0.8392), aligning with prior work by Filev R et al. [21] demonstrating the value of IL-6 combined with radiographic features in optimizing risk stratification in pneumonia patients.

Compared to previous studies, this study advances the field through three key innovations: First, we employed PSM to establish, for the first time, the independent association of NT-proBNP hs-CRP and IL-6 with SCAP while controlling for potential confounders. Second, our analysis dynamically revealed the temporal and synergistic effects of inflammatory markers and CT scores, providing direct clinical evidence supporting the inflammation-injury hypothesis. Third, we propose an integrated multi-indicator model to overcome the limitations of relying solely on individual biomarkers or imaging evaluations. These findings offer novel perspectives for early SCAP intervention, suggesting that NT-proBNP-targeting monoclonal antibodies (e.g., BoxA) or IL-6 receptor antagonists (e.g., tocilizumab) may represent promising therapeutic candidates [22]. However, this study has several limitations, including potential selection bias due to its single-center, retrospective design. Furthermore, the analysis did not incorporate other key inflammatory mediators (e.g., TNF-α, IL-8) for comprehensive profiling, nor did it include longitudinal data tracking the dynamic relationship between inflammatory markers and CT scores. Third, pathogen-specific effects were not analyzed. Bacterial pneumonia may drive higher IL-6 levels than viral etiologies, potentially confounding our results. Future multicenter prospective studies should employ advanced techniques such as single-cell sequencing and spatial transcriptomics to elucidate the spatiotemporal heterogeneity of inflammatory networks and evaluate the clinical efficacy of targeted therapies.

## Conclusion

NT-proBNP/hs-CRP/IL-6 critically contribute to the pathological progression of SCAP by sustaining inflammatory cascades and exacerbating pulmonary injury. Their synergistic correlation with CT findings supports the use of combined biomarker-imaging strategies for disease monitoring, while our integrated predictive model offers enhanced prognostic capability.

## Dodatak

### Availability of data and materials

The data that support the findings of this study are available from the corresponding author upon reasonable request.

### Funding

No funding was received for conducting thisstudy.

### Acknowledgements

Not applicable.

### Conflict of interest statement

All the authors declare that they have no conflict of interest in this work.
